# Gas Flaring in Nigeria: A Multi-level Governance and Policy Coherence Analysis

**DOI:** 10.1007/s44177-023-00045-5

**Published:** 2023-02-14

**Authors:** Godwin O. Aigbe, Lindsay C. Stringer, Matthew Cotton

**Affiliations:** 1 Department of Environment and Geography, University of York, Wentworth Way, Heslington, York, YO10 5NG UK; 2 School of Social Sciences Humanities and Law, Teesside University, Middlesbrough, UK

**Keywords:** Natural gas, Flaring, Venting, Implementation, Climate mitigation, Africa, Climate change

## Abstract

The unnecessary flaring of natural gas impacts public and environmental health, contributes to climate change and wastes fuel resources. Though reducing flaring is an emergent global environmental governance priority, progress has been slow. We assess gas flaring policy in the critical case of Nigeria through multi-level governance (MLG) structure. Our analysis assesses policy coherence (leading to progress in reaching shared goals) and divergence (creating tension and undermining progress) amongst sectors and institutional structures across the supranational, federal, state and local government scales. A combined dataset of documents, stakeholder interviews and expert surveys is analysed using Qualitative Document Analysis (QDA) and content analysis. We identify the principal actors involved, examine the extent of gas flaring awareness and policy coherence across multiple sectors/policy domains, and assess progress towards Nigeria’s national intended contribution and national policy on climate change mitigation. We find that policy coherence around gas flaring, including efforts towards climate change mitigation, has been slowed by political partisanship, poor governance, lack of regulatory compliance, and policy conflict between environmental protection and economic development priorities. Nigeria urgently requires inclusive involvement of stakeholder voices across multiple sectors and scales of local/regional government, the strengthening of federal institutions, a revaluation of economic aspirations through revenue diversification, and leadership that can temper the power of International Oil Companies (IOCs) to exploit the complexity of the MLG structure. These actions would help the government in improving environmental justice outcomes for flaring-affected communities.

**Supplementary Information:**

The online version contains supplementary material available at 10.1007/s44177-023-00045-5.

## Introduction

Flaring of natural gas is common in oil and gas extractive industries, to relieve pressure within pipelines reducing explosion risk, reduce volatile organic compounds through combustion or release waste products from chemical production processes. Flaring involves piping excess gas to a remote (elevated) location and burning the gas in the open air. Though ostensibly gas a matter of routine fossil fuel operations, flaring often occurs due to a lack of economic, regulatory or technical barriers to developing gas markets and gas infrastructure or when it is not feasible to reinject associated gas back into the reservoir (Buzcu-Guven and Harriss 2014; Elvidge et al. 2009; The World Bank Group 2021). Flaring is an environmental management issue due to the release of multiple atmospheric pollutants—including carbon dioxide (CO_2_), carbon monoxide (CO), sulphur dioxide (SO_2_), nitrogen oxide (NO_X_), naphthalene, photochemical oxidants and black carbon particulates (Gobo et al. 2009). Such pollutants detrimentally influence human and environmental health and generate social and economic externalities in affected communities (Osuoha and Fakutiju 2017). The impacts of gas flaring as an environmental justice issue have been historically under-reported due in part to a paucity of measurement, reporting and regulation limited by accurate data. However, recent improvements in accuracy, granularity and availability of remote sensing data on flaring activity (from AVHRR, DMSP-OLS, ATSR, Landsat, MODIS, Soumi-VIIRS and Sentinel-3 SLSTR) in the US and globally (Anejionu 2019; Anejionu et al. 2015; Elvidge et al. 2013) allow improved scrutiny of oil and gas operations, allowing bad practices to be identified and ameliorated through improved environmental governance. Nevertheless, protecting vulnerable communities from gas flaring impacts remains a critical sustainable development challenge, particularly in regions where fossil fuel operations occur under conditions of low regulatory capacity and compliance. Analysis of policy responses to gas flaring activities is, thus, a key research priority for achieving just sustainable transitions in countries with high dependence on domestic oil and gas production for economic and social development. In this paper, we critically assess the policy landscape of Nigerian gas flaring operations.

Gas flaring is a growing area of environmental and cross-cutting policy importance. The need for a flaring reduction strategy was only recently recognised in global climate policy, despite initiatives to reduce fossil fuel extraction and processing emissions. The Global Gas Flaring Reduction Initiative (GGFR) was formally recognised in 2018 following the Paris Agreement and global commitments to keep warming levels below 1.5 °C (UNFCCC 2021). The top gas flaring countries (who are also signatories and active UNFCCC participants) have nevertheless struggled to abate flaring, in part due to the political power of fossil fuel industry lobbying efforts, and the fossil fuel ‘lock-in’ that continues to dominate the economic interests of oil and gas producing nations. Whilst most oil-producing countries have policies towards gas flaring regulation, implementation often differs due to varied policy structures, which in most cases are embedded in complex layers of governance from supranational environmental agreements down to regional and local tiers of environmental planning. Though flaring has become an increasingly visible policy issue globally, it is often addressed through voluntary agreements rather than top-down policy and coordinated action. It is, therefore, necessary to assess gas flaring policy through reference to a conceptual framework of multi-level governance (MLG) to understand the translation of voluntary environmental commitments to national-scale political action in practice, and the influence this has on domestic environmental justice to gas flaring-affected communities.

This paper considers the gas flaring policy in Nigeria. Nigeria is a critical case for such analysis because the continued operation of the oil and gas industry is central to the country’s economic and social development strategy. Nigeria is the largest oil-producing country in Africa, with 37.0 billion barrels of proven crude oil and an even greater 190.4 trillion cubic feet (Tcf) of proven natural gas reserves. Fossil fuels account for > 80% of government revenues, 95% of export receipts, and 90% of foreign exchange earnings (Oladele and Abdul-Azeez 2013; Uwakonye et al. 2006; Watts 2004). The Nigerian Government’s utilisation of natural gas resources is a core aspect of its administrative operations. The government has instigated multiple public–private partnerships for gas development, including the Nigerian Liquified Natural Gas Company, and actively promotes inward investment (e.g. Chevron’s Escravos Gas Utilisation Project for liquified natural gas exports). There is also a strong commitment to social development through gas utilisation, improving policy strategy to improve national electricity access rates through gas-fired power station construction.

Though the national economic and social development strategy emphasises the productive use of gas resources, Nigeria wastes a lot of its resource, remaining in the top seven gas flaring countries globally. The estimated annual flare was 7.83–17.5 billion cubic metres (Bcm) during 2010–2020 (The World Bank Group 2021). Of the 53.6% total CO_2_ emissions contributed by the energy sector in 2000, the Nigerian gas sector accounted for 40.3% (INDC-Federal Ministry of Environment 2014, p. 3 and 37). Between 2010 and 2020, annual natural gas production was between 65.85 and 82.17 Bcm, whilst flaring was between 7.83 and 17.5 Bcm. During 2010–2019, Nigeria produced 750.33 Bcm of natural gas and flared 114.35 Bcm (13%), which, for context, could supply nearly 2 years of the UK’s gas requirements. In 2019, the annual flare increased by 3% (39 million cubic metres (Mcm)) from that of 2018 (NNPC 2020; The World Bank Group 2021). Although flaring decreased 4.9% in 2020, this was attributed to oil price plunges during the COVID-19 pandemic, which led to a corresponding decrease in global oil production. The economic cost of flaring is substantial, despite widely varying estimates. For example, Nigeria is estimated to lose 18.2 million U.S. dollars daily, whilst PWC estimated losses of 761.6 million U.S. dollars in 2018 from flaring (OnlineNigeria.com 2003; PWC 2020), excluding the negative externalities, social, health and environmental harms experienced by residents of major sites of gas production.

From an environmental governance perspective, the situation in Nigeria remains paradoxical and environmentally unjust. Gas flaring was first declared illegal in Nigeria in 1984, though as Akinola (2018) argues, multinational fossil fuel extraction companies continued to treat compliance as a matter of convenience and not of necessity. This attitude has persisted, despite multiple policies, regulations and legal frameworks relevant to gas flaring management at the federal level (Table 1).

**Table 1:** The historical legal frameworks relevant to gas flaring management at the federal level. Source (DPR.GOV.NG 2021; GGFR-World Bank Group 2004; NASS 2020, 2021; NGFCP-DPR 2021)

	Regulation	Year
1	Petroleum Industry Bill (PIB)	2021
2	Petroleum (Drilling and Production) (Amendments) Regulations	2020
3	Petroleum (Drilling and Production) (Amendment) Regulations	2019
4	Flare Gas (Prevention of Waste and Pollution) Regulations	2018
5	The Nigerian Gas Flare Commercialisation Programme (NGFCP)	2016
6	Petroleum (Drilling and Production (Amendment) Regulations	2006
7	Petroleum (Drilling and Production (Amendment) Regulations	2001
8	Petroleum (Amendment) Decree	1996
9	Environmental Legislation-Effluent Limitation Regulations	1991
10	Petroleum (Amendment) Regulation	1989
11	Petroleum (Drilling and Production (Amendment) Regulations	1988
12	Associated Gas Re-injection (Continued Flaring of Gas) Regulations	1985
13	Associated Gas Re-injection Act	1979
14	Petroleum Regulations	1967
15	Petroleum (Drilling and Production) Regulations	1969
16	Petroleum Act	1969

Through the Nigerian Gas Flare Commercialisation Programme (NGFCP), which was explicitly designed to implement the government’s policy objectives to eliminate gas flaring, the Federal Government policy position appears to abate gas flaring whilst the Petroleum Industry Bill (PIB) provides legal, governance, regulatory and fiscal frameworks for the Nigerian petroleum industry, the development of host communities and for related matters (Amaza 2018; NASS 2020; NGFCP-DPR 2021). At the international level, Nigeria ratified the Paris Climate Change Agreement in 2017 and is a signatory to the GGFR principles to end gas flaring by 2030. Correspondingly, Nigeria has committed to tackling climate change through its Intended Nationally Determined Contribution (INDC) and National Policy on Climate Change (NPCC).

Despite escalating policy commitments, flaring activity, particularly across the Niger Delta region, remains high (American Association for the Advancement of Science 2011; Okeke 2019). The Niger Delta constitutes 7.5% of the country’s population and remains the most deeply affected region (Mafimisebi and Ogbonna 2016). Managing trade-offs between fossil-fuel-based economic development and sustainable low-carbon social development remain deeply complex. Although Nigeria functions as a quasi-federalist democracy, the tightly controlled federal structure and personal rule of the presidency is backed by substantial military influence. This creates significant challenges to achieving social justice for the poorest fossil-fuel-producing regions (Ikpe 2000; Leonard and Straus 2003; Takehiko 2010; Yagboyaju and Akinola 2019; Yakubu 2018). Collective legislative action on gas flaring has had minimal or no impact on the poorest Delta communities, meanwhile Nigeria continues to experience a relatively low level of economic development overall (Dartey-Baah et al. 2014; Donwa et al. 2015; Husted and Blanchard 2018; Idemudia et al. 2010; Ncala 2016; The US Department of Justice 2019; Watts 2004). Any justification of environmental harm to Delta-region residents through appeal to the greater public good of oil and gas development tax revenue generation and subsequent social development spending is, therefore, indefensible.

Though the problem of gas flaring in Nigeria is well documented, the contemporary political dynamics of gas flaring governance require further scrutiny, as international pressure to meet climate goals become salient aspects of donor support and aid funding, and pressure mounts from NGOs and environmental activists. We explore the problem through qualitative empirical analysis of gas flaring policy in the context of Nigeria's broader political and institutional structures, policy framework and socio-economic context. We employ multi-level governance (MLG) and policy coherence frameworks to explore how the relationships between different actor perspectives and institutional and policy frameworks operate across different policy sectors in Nigeria. Our research objectives are to:

1. Identify the main actors involved in Nigeria’s MLG system pertaining to oil and gas governance
2. Examine the extent of gas flaring awareness and policy coherence across gas flaring and energy domains
3. Assess the implications for progress towards Nigeria’s national intended contribution and national policy on climate change mitigation.

Our analysis provides insight into environmental governance best practices, through an assessment of policy coherence and implementation across multiple sectors and scales of governance, with recommendations for environmental policy globally.

## Multi-level Governance and Policy Coherence

Multi-level governance (MLG), multi-tiered governance, polycentric governance and fragmented governance are concepts variably used to describe changes to national, international and supranational regimes (Eckerberg and Joas 2004). Within MLG analysis, the emphasis lies upon the interaction of different actors and institutions across multiple scales of policy action, described by Hooghe and Marks (2001) as authority diffusion from the central government up to the supranational level, down to subnational jurisdictions and sideways across public/private networks. Hooghe and Marks (ibid.) identify two distinct visions: MLG Type I and MLG Type II. In Type I MLG, the dispersion of authority is relatively stable, with no jurisdictional overlap and limited levels. Type II MLG is a complex structure with jurisdictional overlaps and is usually situated within Type I jurisdictions. These jurisdictions are flexible, temporary and characterised by their interchangeability.

Policy coherence implies a relationship and consistency between policy goals, policy instruments, implementation and outcomes (Huttunen et al. 2014; Mickwitz et al. 2009). Coherence advances cooperation between and within complex policy domains to realise policy objectives that are collectively agreed upon and recognised (Nilsson et al. 2012). To facilitate the implementation of gas flaring policies, individual sectoral policies need to be consistent with one another. Of note is the coherence between policy domains pertaining to energy, economic development, and mitigation and adaptation to climate change. For example, policies that encourage energy export for economic growth, assuring local environmental and public health compliance and growing tax revenue collection for domestic social development, may provide a package of self-reinforcing sustainable development objectives or else may produce internal conflict with one or more policy objectives undermining the achievement of the others. Conflicting goals and rules present significant challenges to policy coherence in an MLG system (Sandström et al. 2019). Understanding how MLG and policy coherence operate in concert is the subject of our empirical analysis.

## Methodology

Our four-stage Qualitative Document Analysis (QDA) approach was used to undertake a horizontal-level policy coherence analysis. We follow a four-step QDA model (identified in Altheide et al. 2008), as described below:

1. Set the criteria for document selection
2. Select and obtain the relevant documents
3. Perform Qualitative Document Analysis (QDA)
4. Validate through interview and survey data collected from stakeholders within the sector.

*Step 1: Setting the Criteria for Document Selection*

For the analysis of academic literature, a Google Scholar search was performed, combining the text words “gas flaring” AND/OR “gas venting, in Nigeria”, restricting the search to English language journal articles.

Official government policy documents were sampled. Inclusion criteria in selecting documents were those pertaining to: oil and gas—exploration, extraction or development and related sectors that provide reference to gas flaring; and climate change and greenhouse gas emission reduction strategies. The sampling frame covered publication years 2000–2020.

As some documents include oil spills and other environmental hazards, only segments related to gas flaring were considered. Key sectors contained within the sectoral adaptation and mitigation programmes were identified from the National Policy on Climate Change (NPCC) 2013 document.

*Step 2. Select and Obtain the Relevant Documents*

Sectoral government policy documents were obtained through an internet search, combining the search terms “gas flaring policies and/or regulations” and/or “Nigeria policy documents” with subject headings “Nigeria gas flaring, and/or NDP and/or INDC, and/or climate change”. The corpus was restricted to English language publications. Relevant government websites displayed during the initial search were further searched to locate specific sector policies. Table 2 details the final documents obtained and selected for analysis.

**Table 2:** Documents that make up the sample for Qualitative Document Analysis

Sector	Policy document title	Year published
Energy policy	National Energy Policy	2003
Climate Change	National Policy on Climate Change	2013
Intended Nationally Determined Contribution (INDC)	Nigeria's INDC	2015
Gas policy	National Gas Policy	2017
National Action Plan (NAP-SLCPs)	National Action Plan to reduce Short-Lived Climate Pollutants	2018
National Development Plans (NDP)	Nigeria Economic Sustainability Plan	2020

*Step 3: Qualitative Document Analysis*

Systematic policy coherence analysis of the selected sectoral policy documents was performed across sectors. Documents were analysed using content analysis (e.g. England et al. 2018; Stemler 2001) comprising four stages. The first stage (3a) involved counting and scoring each keyword. Each of the policy documents was assessed to ascertain language pertaining to gas flaring and venting, then its level of conceptual linkage to other policy domains, and the prevalence of mitigation strategies and measures contained within the policy framework. Data were coded thematically using NVivo software and utilised to locate each of the gas flaring concepts within the sector’s policy documents for dominant strategies grouped into four main themes: (a) gas flaring and venting, (b) energy (c) national gas and energy inter-sector alignment for climate change mitigation (d) climate change mitigation. Each sector policy was reviewed, and the number of times each concept was mentioned was recorded. Keywords “natural gas”, “flaring”, “climate”, “venting”, “gas flaring”, “climate change”, “mitigation”, and “climate mitigation and financing” were searched for within the documents. The semantic and discursive context of the gas flaring-related concepts within the sentence, phrase, or paragraph in which they appeared was then assessed.

In the second stage (3b), direct content analysis assessed the level of coherence of each policy document using the keywords to locate the sentence or paragraph where they were used within each policy document. This involved thorough reading of each document to ascertain the specific background context and insight into the government’s strategies, particularly how these keywords were prioritised within the strategic plans. Keywords were then grouped and re-organised into themes based on relationships focussing on climate change policy strategies and INDC plans, facilitating cross-comparison.

The third stage (3c) involved checking for coherence using keywords. The number of times each of the keywords was mentioned formed the first part of assessing awareness regarding gas flaring. Each mention of the various gas flaring and venting-related concepts was assessed according to how they were used in the paragraph. Scores were applied to the level of coherence, ranging from 0 (no coherence) to 3 (high coherence) for each policy document concerning awareness of each of the nine gas flaring concepts and the extent of relevant strategies using a categorisation matrix (e.g. Elo and Kyngäs 2008) (Table 3).

**Table 3:** Scoring criteria to assess coherence and their definitions

Type of coherence	Description of coherence	Score
High coherence	Policy document aligns strongly across national gas policy, energy and climate change statements. Policy dedicates a specific attention to both gas flaring and venting mitigation and energy inter-sector alignment concerning climate mitigation. It includes a range of detailed complementary measures and plans to achieve coherence	3
Partial coherence	Whilst the policy document supports both the national gas and energy inter-sector alignments in relation to climate change mitigation, inadequate details on associated measures are provided, and it is unclear how it could be achieved. Also, limited activities and plans are incorporated but lack comprehensive detail	2
Limited coherence	Policy document supports national gas and energy inter-sector alignment concerning climate mitigation (particularly in the form of general statements). However, no details on activities or plans are provided	1
No coherence	Policy document lacks evidence of coordination or alignment in sectoral statements	0

The keywords were then used to search each policy document to assess how and to what extent they described the same issue. Average coherence scores were generated for the various documents.

The fourth stage (3d) considered the level of coherence between sectors. This involved careful review of each document to identify when each of the other sectors was either explicitly mentioned or implicitly considered by averaging the two values ascribed to each policy document as calculated in stage three.

*Step 4. Validation and Finalisation Through Qualitative Interview and Survey Responses*

Validation and finalisation (steps 3 and 4 of the QDA) comprised stakeholder interviews with actors involved in gas flaring, including climate change mitigation and adaptation action in Nigeria. Attempts were made to schedule interviews with the five federal institutions responsible for gas flaring management in Nigeria, but tight political controls meant participation was consistently declined. Furthermore, 30 individuals, employees of these federal ministries, institutions and agencies who were contacted declined our invitation due to “instruction from the top” not to participate. Consequently, seven experts (four representatives from environmental NGOs and advocacy groups and three environmental campaigners) were interviewed, whilst an industry experts survey targeted people with a PhD in oil and gas and related fields and policy experts.

We employed purposive sampling to identify experts for the survey and utilised a snowball sampling technique to recruit the other experts. We contacted 59 experts through e-mail, and 23 replied and completed the survey. Interview and survey respondents’ roles or connections with their organisations were anonymised. NVivo software was utilised in coding and analysing the validation data according to sectoral themes.

## Results

First, we present key findings from our literature analysis of Nigeria’s MLG system pertaining to oil and gas governance. Second, we show the extent of gas flaring awareness and policy coherence across the policies of different sectors, and finally, we examine the implications for progress towards Nigeria’s INDC and NPCC.

### Nigeria’s MLG System Pertaining to Oil and Gas Governance

**Fig. 1:**
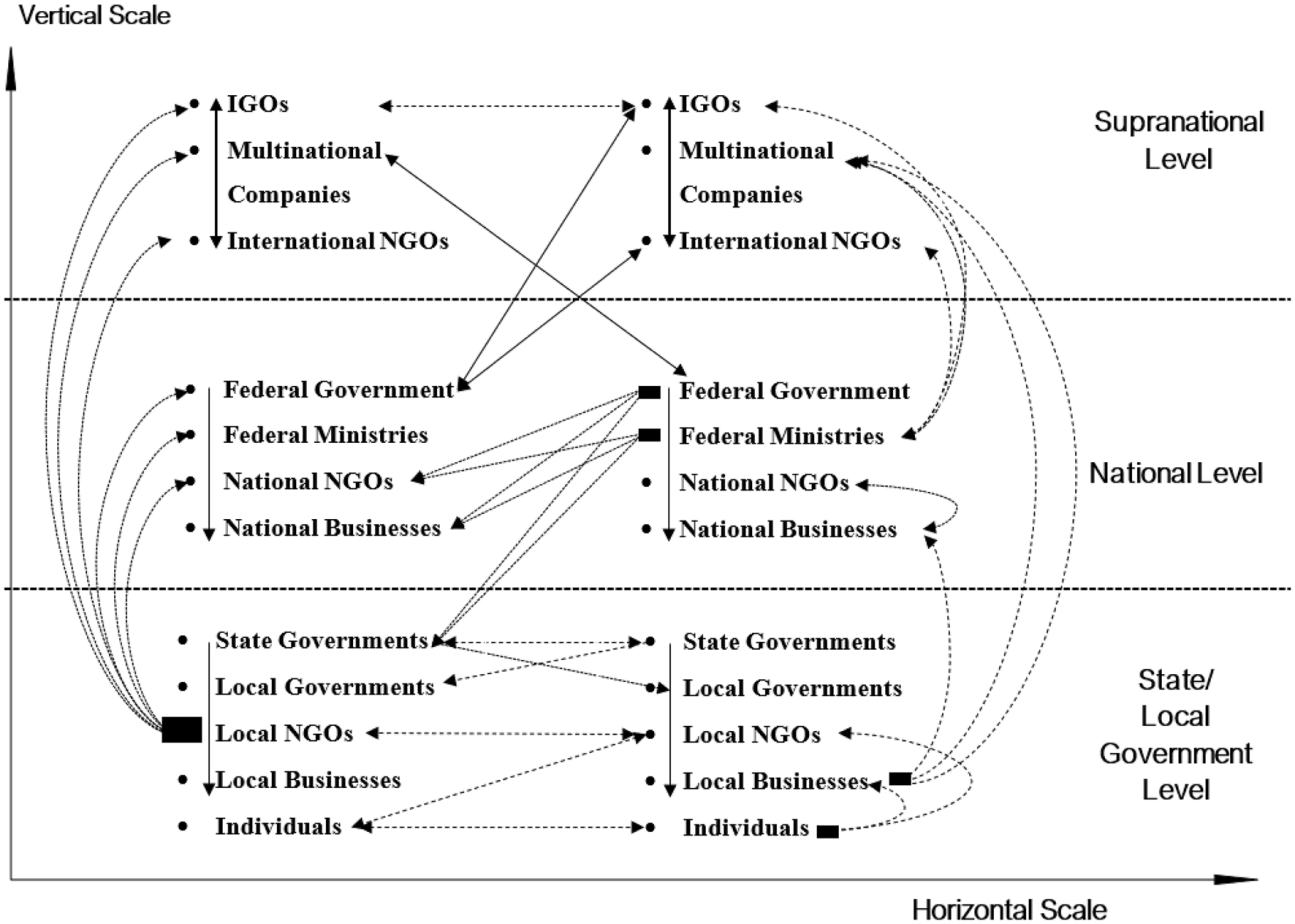
Horizontal and vertical dimensions of the Nigerian gas flaring management within a Type II MLG system. Adapted from Andonova and Mitchell (2010, p. 258). Dotted arrows identify interactions where the action occurs involving many political actors operating across vertical and horizontal scales of jurisdictions, space, issues and organisational domains. *IGO* Inter-Governmental Organisations, *NGOs* Non-Governmental Organisations

Following independence from British colonial rule in 1960, Nigeria began operating as a Federal Government system. The Nigeria constitution of 1999, as amended, provides the basis for all laws and legislation at the highest level. Nigeria currently has 36 states, a federal capital territory (FCT) and 774 local government areas as a federation. States are not autonomous but depend on federal allocation and the 13% derivation from oil and gas producing states (The Federal Government of Nigeria 2008; World Bank 2002). Vertically, the federation account's distribution is 48.50%, 26.72%, 20.60% and 4.18% to the federal, state and local governments and centrally controlled special fund (Ohiomu and Oluyemi 2019; Suberu 2019). On paper, Nigeria’s governance structure is deeply rooted in federalism, relatively stable and with no jurisdictional overlap, with power-sharing amongst a limited number of authorities operating across three levels of governance (federal, state and local) (e.g. Hooghe and Marks 2001 and Fig. 1). In practice, diverse sectors and government agencies function under a tightly controlled centralised governance structure—this, in turn, a legacy of successive military dictatorships structuring the institutional fabric of Nigerian politics.

**Fig. 2:**
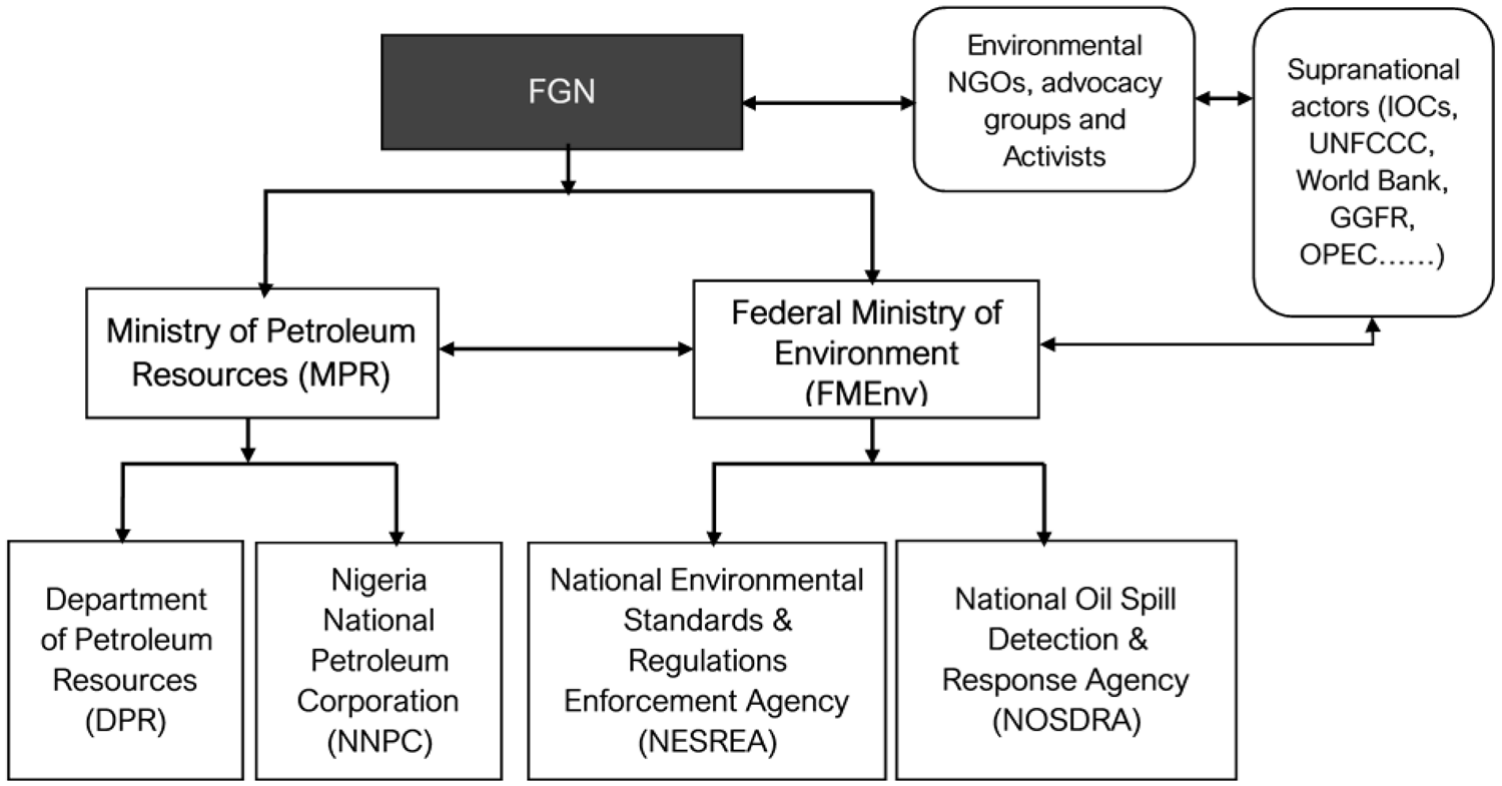
Federal institutions, environmental NGOs, advocacy, activists and supranational actors involved in gas flaring and venting. *FGN* Federal Government of Nigeria

The Federal Ministry of Petroleum Resources (MPR) and Federal Ministry of Environment (FMEnv) are responsible for managing gas flaring and other environmental issues through parastatal organisations that wield indirect political authority (namely: the Department of Petroleum Resources (DPR), Nigerian National Petroleum Corporation (NNPC) and the National Environmental Standards and Regulations Enforcement Agency (NESREA)) (Fig. 2). These federal institutions are responsible for formulating policies and regulations on environmental issues, including gas flaring. The historical context of Nigeria’s gas flaring management is important because the parastatal organisational and administrative structure underpins Nigerian MLG and plays a principal role in policy implementation and public service delivery.

The first statutory agency established to regulate the petroleum industry was the DPR. In 1971, the Nigerian National Oil Corporation (NNOC) was established to manage the commercial and operational activities in the oil industry, whilst DPR under the Federal Ministry of Mines and Power exercised statutory guidance and industry control. The DPR was adapted into the Ministry of Petroleum and Energy in 1975 and later renamed the MPR. In 1977, the MPR and the NNOC were consolidated to create the NNPC. However, the petroleum inspectorate was still an integral component of the NNPC and was mandated to regulate the petroleum industry. The MPR was re-established in 1985, whilst the petroleum inspectorate remained within the NNPC. The NNPC was re-organised in 1988, and the petroleum inspectorate was moved to the MPR as the technical division and renamed the DPR (DPR 2020; FMEnv 2020; MPR 2019; NESREA 2020; NNPC 2020; Olawuyi 2015; Otiotio 2013).

The MPR is the main federal executive organ responsible for articulating and implementing petroleum resources policies, maintaining standards, monitoring quality and quantity and regulating industry practices. The minister has discretionary powers to grant flaring. The DPR and the NNPC are parastatals under the MPR. Through the MPR, the DPR is responsible for overseeing all the activities of all companies engaged in oil and gas production. It ensures and facilitates compliance with the applicable laws and regulations, including enforcement of safety and environmental regulations, and advises the government and relevant agencies on technical and policy issues (MPR 2019; DPR 2020; NNPC 2020).

The Federal Ministry of Environment (FMEnv) was established in 1999 to protect and improve Nigeria's water, forest, land and air by formulating, coordinating and implementing environmental policies and programmes, prescribing standards, and formulating and enforcing environmental regulations and laws. NESREA and the National Oil Spill Detection and Response Agency (NOSDRA) are parastatals under the FMEnv. NESREA was established by the NESREA Act of 2007 and empowered to enforce all environmental laws, guidelines, policies, standards and regulations and ensure compliance with international treaties, conventions, protocols and environmental agreements (MPR 2019; DPR 2020; FMEnv 2020; NESREA 2020).

International Oil Companies (IOCs) and organisations such as the United Nations Framework Convention on Climate Change (UNFCCC), World Bank, GGFR and OPEC are also involved in gas flaring management. However, power is unevenly distributed across the governance system. Though several different parastatal organisations are involved in shaping the overall gas flaring management structure in Nigeria, political power still follows the conventional top-down quasi-federal structure. Analysis of the expert survey data indicated that 45% of those surveyed considered that the IOCs weaken policy implementation due to their influence on government policies to help them align with their business goals, whilst the 36% who agreed that the IOCs strengthen policy implementation was mainly working in technical fields (engineering) and employees of the IOCs. The remainder considered the influence of the IOCs has a neutral influence on gas flaring policy implementation. Interviewees underscored these findings. One respondent stated:

*I have described the oil companies in Nigeria as a country within a country. The oil companies are high key players in the politics of Nigeria and the economy of Nigeria. The oil companies do work together with the Federal Government* (Environmental Campaigner 3).

A second respondent noted that:

*If you tell Shell to stop flaring today, then there is no need to produce oil then. The oil companies know that Nigeria’s government is not ready to stop gas flaring* (NGO representative 4).

The first factor that shapes the Nigerian MLG structure is the amended 1999 Constitution. The Nigerian governance structure operates within a Type II MLG system, and its structure has created loopholes, overlapping policy domains, fragmentation, and enclaving (“a country within a country”) whereby extractive industries operate in a self-governing manner outside of national regulatory jurisdiction (Ackah-Baidoo 2012). The Federal Government formulates and implements gas flaring policy, but the role of non-state organisations (specifically IOCs) creates the conditions for a complex Type II MLG structure (Ikpe 2000; Yagboyaju and Akinola 2019; Yakubu 2018). Notwithstanding, under Part II of the Nigeria constitution, section 4 (1) Sect. 3 vested the legislative powers of the Federal Republic of Nigeria in the National Assembly to make laws for the Federation concerning any matter included in the exclusive legislative list. That provision makes it extremely difficult for the regional state and local governments to interact directly with the Federal Government, including on issues concerning gas flaring, even though the pollution caused occurs primarily within the various states’ jurisdiction within the Niger Delta. States have little or no power to control gas flaring because the policy issue belongs to the exclusive list. Therefore, they are reliant upon federal-scale top-down policy implementation and enforcement through parastatal actors, and hence lack a ‘voice’ in the processes of good environmental governance (World Bank 2002; The Federal Government of Nigeria 2008).

The second factor that shapes MLG is legislative change. The Federal Government has introduced various plans for new laws on gas flaring. At the federal level, all the hydrocarbon legislation (Aye and Wingate 2019; Udok and Akpan 2017; The World Bank Group 2004) is relevant to gas flaring management in Nigeria. Although these laws were introduced to mitigate gas flaring and venting, no proper consultation or discussion with interested parties was considered since there is a reoccurring concern of low stakeholder engagement in Nigeria’s oil and gas management (Ayotunde 2016; Emoyan 2010; Enuoh 2015), particularly of state governments and host communities. Hence, on paper, these laws may have been carefully formulated, as numerous interviewees noted:

*One thing is wanting to have a policy, and it is another thing if you genuinely want the policy to work"* (NGO representative 3).

*The fact that these agencies exist does not necessarily mean that they are effective. DPR is controlled at the federal level. Individuals who own oil wells and oil companies mostly are not from the region"* (Environmental Campaigner 3).

Validating the complex and fragmented Type II MLG system (Fig. 2) with unclear leadership in the various authorities concerned with gas flaring management, analysis of the expert survey data indicated that 23% of those surveyed agreed that the local and state government priorities and politics concerning flaring match those of the national government, 45% considered that they do not match, whilst 32% answered ‘unsure’. Interviewees underscored these findings:

*To the best of my knowledge, I do not think that Federal Government involves all the possible stakeholders in gas flaring and in deciding whether to flare. It is usually the Federal Government, IOCs, and other companies that sit down and discuss and come up with rules of flaring* (Environmental Campaigner 2).

*There is hardly any involvement, and it is the lack of involvement that has led us to where we are today, the agitation, the activism in the region to complain about gas flaring to complain about, about injustice* (Environmental Campaigner 3).

We further asked survey respondents how well they consider policy goals and stakeholder preferences fit together. Again, 45% said that the policy goals and preferences do not fit together, 20% considered that they do fit and 35% were undecided.

The third factor shaping MLG and, subsequently, policy coherence and implementation is the administrative structures (Figs. 1 and 2). Sectors and government agencies function under a tightly controlled governance structure, but federal institutions and agencies tasked with gas flaring management lack the autonomy to function effectively as monitoring and reporting bodies (Ako and Olawuyi 2017). At the federal level, restructuring of ministries and government agencies across electoral cycles every 4 years has created barriers to long-lasting policy commitments (Fourchard 2011; Kolawole 2020; Zovighian 2018). One interviewee noted:

*There have been different governments. This one comes in, changes policies, and even within the same government, you have a somersault in policies. We have not had any strategic push to develop a plan* (Environmental Campaigner 2).

The Federal Government also exercises strict political control over ministries/institutions and parastatal agencies located in the political centre in Abuja. However, the physical distance (Abuja and the Niger Delta region is approximately 550 km apart) creates a political geography whereby the federal capital territory positions the Niger Delta region as a sacrifice zone to meet other national policy goals (de Souza 2021; Ogwu 2012; Unah and Iruoma 2021). As an interviewee noted:

*The government concentrates power in Abuja, very far away from where solutions need to be. Until we diversify and see the need for other economic sectors to boost our economy, the people of the Niger Delta region or where the oil is being extracted from will become the sacrificial lamb* (NGO representative 4).

### Gas Flaring Awareness

Table 4 summarises awareness of gas flaring, venting and related concepts. Despite overlaps between, for example, natural gas, flaring and gas flaring, the coherence scores of each concept were equally weighted for this research. Across the policies analysed, eight gas flaring and venting-related concepts were identified to generate the themes in the analysis. Notably, none of the documents analysed mentioned venting. The National Action Plan to reduce Short-Lived Climate Pollutants (NAP-SLCPs 2018) and Intended Nationally Determined Contribution INDC (2015) documents demonstrated the highest level of awareness, with scores of 2.6 and 2.4, respectively.

**Table 4:** Coherence of policy documents and relevant keywords relating to gas flaring and venting concepts (coherence score 0–3)

	National Energy Policy (2003)	National Policy on Climate Change (2013)	Nationally Determined Contribution (2015)	National Gas Policy (2017)	National Action Plan (NAP-SLCPs) (2018)	National Economic Sustainability Plan (2020)
Natural Gas (Score)	28 Mentions (3)	3 Mentions (1)	5 Mentions (2)	55 Mentions (3)	26 Mentions (3)	6 Mentions (2)
Flaring (Score)	15 Mentions (3)	3 Mentions (1)	13 Mentions (3)	86 Mentions (3)	67 Mentions (3)	1 Mention (1)
Climate (Score)	2 Mentions (0)	81 Mentions (3)	122 Mentions (3)	3 Mentions (1)	172 Mentions (3)	0 Mention (0)
Venting (Score)	0 Mention (0)	0 Mention (0)	0 Mention (0)	0 Mention (0)	0 Mention (0)	0 Mention (0)
Gas Flaring (Score)	9 Mentions (3)	3 Mentions (2)	7 Mentions (2)	27 Mentions (3)	46 Mentions (3)	1 Mention (1)
Climate Change (Score)	0 Mention (0)	69 Mentions (3)	86 Mentions (3)	2 Mentions (1)	57 Mentions (3)	0 Mention (0)
Mitigation (Score)	5 Mentions (1)	23 Mentions (3)	31 Mentions (3)	1 Mention (0)	66 Mentions (3)	3 Mentions (0)
Climate Mitigation and Financing (Score)	0 Mention (0)	4 Mentions (2)	9 Mentions (3)	0 Mention (0)	6 Mentions (3)	22 Mentions (0)
Mean Score	**1.3**	**1.9**	**2.4**	**1.4**	**2.6**	**0.5**

### Policy Coherence Across Sectors

The National Gas Policy (2017) recognises gas flaring, including its mitigation strategies. The document proposed a clear policy, institutional, legal and regulatory framework, including gas policy; gas legislation; regulatory authority; secondary legislation (regulation) to end gas flaring and address environmental issues. The National Energy Policy (2003) is old but still the most recent energy policy and details gas flaring, including mitigation strategies. The document proposed several strategies to eliminate the flaring of associated gas by 2008 by encouraging oil-producing companies to gather and utilise associated gas and imposing appropriate and effective penalties to discourage gas flaring. The most frequently mentioned strategies for gas flaring mitigation in the energy policy document include gas utilisation, flaring penalties, gas infrastructure, incentives, governance and regulations. The same issues identified in the 2003 policy were repeated in other documents. For example, one of the energy policy objectives was “*To eliminate the flaring of associated gas by 2008*” (NEP 2003). NPCC (2013, p. 2) noted the importance of: “…*supporting ongoing initiatives to gradually eliminate gas flaring*”. The Nigeria Economic Sustainability Plan 2020 is the current National Development Plan (NDP 2020) anchored in Vision 20:2020: The Federal Government’s economic growth plan and the Nigeria Economic Transformation Blueprint (2009). Although the NDP is a developmental document that should emphasise sectoral strategies, it provided no evidence of a link between flaring policy and climate change mitigation action. However, it does mention The National Gas Flare Commercialisation Programme, although this has not yet commenced.

Whilst the NPCC recognises gas flaring and detailed strategies, there were instances where the importance of inter-sectoral linkages to achieve mitigation was included. Several gas flaring and energy mitigation strategies were identified in both INDC and NDP documents. The NAP-SLCPs (2018) detailed abatement measures to eliminate gas flaring by 2020, explicitly stating that “*Elimination of gas flaring and recovery and utilization of vented associated gas*” (NAP-SLCPs 2018, p. 51) was of high priority within measures linked to the NGFCP document’s current plans. However, the NGFCP is yet to start in 2021. Despite gas flaring and energy sectoral policies being mentioned in most of the documents analysed, detailed strategies were presented in the NAP-SLCPs and INDC documents. The INDC also mentioned: “…*work towards ending gas flaring by 2030*” (INDC 2015, p. 2). The National Gas Policy (2017) stated that: “*The commercialisation of flared gas for supply into the domestic market is a high priority strategy for the Government in achieving the national mandate for flare-out by 2020*” (NGP-MPR 2017, p. 62), whilst the NAP-SLCPs (2018, p. vi): aspired to see: “*100% of gas flaring eliminated by 2020*”. These objectives clearly commit to a sequential reduction in flaring, though in practice, there has been an increase (NNPC 2020; The World Bank Group 2021).

In terms of the coherence of documents around climate change mitigation across sectors, the NPCC (2013), NAP-SLCPs (2018) and INDC (2015) scored the highest, each with a coherence score of 3 (Table 5). When considering the extent of coherence in national policies across gas and energy sectors and with climate change mitigation goals outlined in national policy on climate change, results indicate the NAP-SLCPs (2018) is most coherent with a mean score of 2.75 whilst NPCC (2013) and INDC (2015) both scoring 2.5. Other documents analysed fall between limited and partial coherence. Newer versions of each policy are not incremental iterations of the previous version, possibly due to the need to use the document to secure international funding, as was found in Malawi by England et al. (2018). For example, whilst the original vision and mission statement of the national gas policy centred primarily upon economic derivatives, both the 2014 and 2015 INDC reports emphasised the need for international finance as a prerequisite to achieving the INDC plans. The National Gas Policy (2017, p. 32) seeks to: “…be an attractive gas-based industrial nation… […] … and developing a significant presence in international markets” and to “…move Nigeria from a crude oil export-based economy to an attractive oil and gas-based industrial economy”. Similarly, the INDC (2014, p. 5–6) notes that: “Unfortunately, the economic situation of the country Nigeria makes it challenging for the government to allocate sufficient funds for climate change programmes… […] …International finance and investment, technology and capacity building will be needed to achieve the ambitious intended contribution”.

**Table 5:** Coherence of policy documents for key themes and adaptation keywords (score 3 = high coherence; 2 = partial coherence; 1 = limited coherence; 0 = No coherence)

	National Energy Policy (2003)	National Policy on Climate Change (2013)	Nationally Determined Contribution (2015)	National Gas Policy (2017)	National Action Plan (NAP-SLCPs) (2018)	National Economic Sustainability Plan 2020
Gas flaring	Details gas flaring and includes mitigation strategies. But no mention of venting throughout the document (2)	Recognise gas flaring but no mention of venting throughout the document. But no details on associated mitigation measures provided (1)	Details gas flaring and includes mitigation strategies. But no mention of venting throughout the document (2)	Details gas flaring and includes mitigation strategies. But no mention of venting throughout the document (2)	Details gas flaring and includes mitigation strategies. But no mention of venting throughout the document (2)	Recognise gas flaring. However, no details on associated mitigation measures provided (1)
Energy	N/A	Policy recognises climate change variability with detailed strategies with prioritisation (3)	Policy recognises the importance of energy in mitigation of climate change with detailed strategies with prioritisation (3)	Policy recognises gas flaring and includes mitigation strategies. But no mention of venting throughout the document (2)	Policy recognises the importance of improved energy efficiency in climate change mitigation (3)	Policy recognises the importance of the energy sector with few details and strategies included (2)
National gas, and energy inter-sector alignment for climate change mitigation	Document recognises gas flaring inter-linkage and provided some strategies to achieve integration (2)	Document recognises the importance of inter-sector linkages and includes a number of approaches to achieve integration (3)	Document recognise importance of inter-sector linkages and includes a number of approaches to achieve integration (2)	No explicit reference to inter-sector alignment, few references plans to establish linkages (0)	Document recognises the importance of inter-sector linkages and includes detailed associated strategies (3)	General statement of inter-sector linkage but no mention of associated strategies (1)
Climate change mitigation	Few evidence suggesting climate change mitigation (1)	Details potential climate change and a few specific mitigation strategies (3)	Details potential climate change and several specific mitigations strategies (3)	No evidence suggesting climate change mitigation strategies included (0)	Details potential about climate change mitigation strategies (3)	No evidence suggesting climate change mitigation awareness (0)
Mean Score	1.3	2.5	2.5	1.0	2.75	1.0

Scoring criteria to assess coherence. Adapted from Le Gouais and Wach (2013); England et al. (2018). Coherence = 3: The policy document shows a high level of awareness of gas flaring and venting concept. The document gives specific attention to gas flaring and venting. It includes various and comprehensive complementary measures. Partial coherence = 2: The policy document shows general awareness of gas flaring and venting. Despite recognising the importance of gas flaring, however, there are few details and measures included within the policy document. Limited coherence 1: The policy document recognises gas flaring and venting. However, no details on associated measures are provided. There is no coherence = 0: There is no evidence in the policy document to suggest gas flaring.

Assessment of the coherence of policies relative to one another revealed the highest scores for the NAP-SLCPs (1.84), NPCC (1.75) and INDC (1.75). However, the highest possible score was 3, indicating that the level of coherence concerning gas flaring in sectoral policies is between limited and partial coherence (Table 6). Expert survey findings validated this. All respondents agreed that incoherent policies and inconsistent as well as conflicting regulatory frameworks were problematic, with implementation challenges marred by corruption and poor governance. Of the 23 respondents, 67% considered incoherent policies might create difficulties in executing the gas flaring reduction strategy, and 59% strongly agreed that an inconsistent and conflicting regulatory framework might create difficulties in executing the gas flaring reduction strategy.

**Table 6:** Coherence of policy documents (3 = high coherence; 2 = partial coherence; 1 = limited coherence; 0 = no coherence

	National Energy Policy (2003)	National Policy on Climate Change (2013)	Nationally Determined Contribution (2015)	National Gas Policy (2017)	National Action (NAP-SLCPs) Plan (2018)	National Economic Sustainability Plan (2020)	Total
National Energy Policy (2003)	NA	1.88	1.88	1.13	2	1.13	1.34
National Policy on Climate Change (2013)	1.88	NA	2.5	1.75	2.63	1.75	1.75
Nigeria's INDC (2015)	1.88	2.5	NA	1.75	2.63	1.75	1.75
National Gas Policy (2017)	1.13	1.75	1.75	NA	1.88	1.0	1.25
National Action Plan (NAP-SLCPs) (2018)	2	2.63	2.63	1.88	NA	1.88	1.84
National Economic Sustainability Plan (2020)	1.13	1.75	1.75	1	1.88	NA	1.25
Mean Score	1.34	1.75	1.75	1.25	1.84	1.25	9.18

### Implications for Progress Towards Nigeria’s National Intended Contribution and National Policy on Climate Change Mitigation

Despite climate change mitigation being mentioned once and recognised in general statements in the National Gas Policy (2017), there was little detail concerning gas utilisation as a climate change mitigation strategy. However, specific details of intended measures and detailed strategies describing climate change mitigation were included in the NPCC, INDC and NAP-SLCPs. The National Energy Policy (2003) mentioned mitigation five times, though with little detail on strategy or implementation. Most of the mitigation measures in the National Gas Policy and Energy Policy documents were replicated in the INDC and NAP-SLCPs. For example, the National Gas Policy (NGP-MPR 2017) stated that the country would: *“End gas flaring and address environmental issues”.* The same measure was found in the National Energy Policy (NEP 2003), which strived to: “…*eliminate the flaring of associated gas by 2008”* and (NAP-SLCPs 2018) sought: “…*elimination of gas flaring” and “100% of gas flaring eliminated by 2020”.* Whilst mitigation was mentioned three times in the NDP document, it was in the context of economic goals to: *“mitigate the effects of a deep recession”.* This raises questions about whether the government is genuinely committed to ending gas flaring due to the economic benefits from oil and gas revenues; hence, the repeated extension of the target date. Expert survey data indicated that 53% agreed that economic policies dominate environmental policies and gas flaring concerns; 33% disagreed, and 14% were undecided. Interviewee responses corroborated this, for example:

*The Nigerian economy is driven by an economic goal, with 90% of foreign earnings coming from oil and gas, while the government’s attention is more focused on the economic benefits (earnings of that sector) than the environment. From what I see on the ground, economic derivatives or benefits weigh higher in the ranking of government interest than how the people feel. Hence, the Federal Government refused to sign the Petroleum Bill, and they kept shifting the goalpost* (NGO representative 3).

Despite detailing the policy relevance of climate change and several specific mitigation strategies in the INDC, NPCC and NAP-SLCPs, it is unclear how such strategies can be achieved in practice within the entrenched fossil capitalist economic model in Nigeria. Climate change concepts in policy documentation may, therefore, be used to secure international climate finance funding rather than reflecting a desire for serious economic transformation. For example, the INDC (2015) stated that Nigeria would: “Work towards ending gas flaring by 2030”. However, the NAP-SLCPs (2018) stated that 100% of gas flaring would be eliminated by 2020. Repeated shifting of the target dates makes overall goals unclear and appears to override any progress recorded towards Nigeria’s INDC and national policy on climate change mitigation whilst exacerbating the violent conflicts attributed to the ostensibly visible environmental injustices in the Niger Delta (Ako and Olawuyi 2017; Gonzalez 2016).

## Discussion and Conclusion

This study shows that the main actors involved in Nigeria’s MLG system pertaining to oil and gas governance operate under a fragmented Type II MLG structure with unclear leadership in the various authorities concerned within a tightly controlled and top-down quasi-federal governance regime. The lack of political will to end gas flaring (Akinola and Wissink 2018; Benson 2020; Iornumbe 2019; Olujobi 2020) is also attributed to the Federal Government’s and other stakeholders’ continued economic interest in fossil fuel extractivism.

Nigerian energy and environmental politics are incremental rather than transformative due to the challenges of partisan federal politics. The current and successive military government regimes are characterised by dictatorship, rent-seeking and corruption and personal rule. These factors concentrate political power in Abuja in a ‘top-down’ policy framework, failing to address environmental injustices in the Niger Delta. Political elites insufficiently recognise the adverse impacts of gas flaring suffered by host communities yet continue to benefit from gas production. The concentration of political power further diminishes state governors' capacities to influence gas flaring outcomes from within the fragmented MLG Type II system. Quasi-federalism restricts regional/state stakeholders from speaking to legislative change, removing their political voice (Ako and Olawuyi 2017; Gonzalez 2016) as their power as heads of the federating units within their state's jurisdictions is curtailed. This situation has also made it expedient for the government to coerce individuals and heads of government’s institutions/agencies to guarantee policies cohere across sectors on paper, even though the implementation impacts of environmental regulation are weak.

Government ministry and agency restructuring across electoral cycles every 4 years has created barriers to long-lasting policy commitments, leading to “somersault policies”. Also, the Federal Government’s strict political control over ministries/institutions and parastatal agencies allows the federal capital territory to position the Niger Delta region as a *sacrifice zone* (de Souza 2021; Ogwu 2012; Unah and Iruoma 2021) to meet other national policy as well as personal and partisan policy goals. These influences strongly shape the MLG structure and policy coherence, resulting in a lack of coordinated action and political will to end gas flaring.

Analysis of Nigeria’s INDC and policy on climate change mitigation suggests policy coherence is driven by a need to secure international climate finance. The Federal Government's heavy involvement in gas flaring management is contextualised by continued economic interest in maintaining the status quo within the gas sector, with environmental performance a subsidiary concern to wealth generation. This creates an implementation gap between the legislation on flaring and the enforcement of environmental protection measures that would adversely impact the IOCs and, by extension, the Federal Government. Relevant regional and state-level authorities and stakeholders remain excluded from decision-making, whilst those charged with enforcement are hesitant to carry out their respective functions relating to gas flaring management and regulatory practice. Although the Federal Government instituted the NGFCP in 2016 to end gas flaring, it faces additional concerns that may further widen the implementation gap: the deliver-or-pay agreements (a requirement by licensees to guarantee the production of definite volumes of gas and be liable to pay or compensate for any shortfall where the contracted volume cannot meet the requirements); the rights of contractors under Production Sharing Contracts (PSC) with the NNPC joint ventures; the exclusion of benefits for some critical stakeholders with the largest investments (the oil companies) from the NGFCP programme; and the huge investments required to develop new infrastructure for flare gas collection and supply (Amaza 2018; NGFCP-DPR 2021). Although 200 bidders from 800 bids have been shortlisted, the initiative’s economic benefits cannot be evaluated currently as the project is yet to commence formally. The implementation gap, thus, underscores five principal concerns summarised in Table 7.

**Table 7:** A summary of concerns and lack of action

1	The implementation of gas flaring reduction suffers from policy incoherence from successive goal conflicts where gas flaring and other environmental and climate change mitigation policies give way to economic goals. Whilst the Federal Government exerts tight control across the institutions and agencies, the federal institutions involved with gas flaring do not have sufficient autonomy over gas flaring regulation enforcement to hold the national authorities and other federal ministries accountable
2	There is a lack of coordinated horizontal gas flaring and climate change mitigation governance and policy process across all sectors. The analysis of sectoral policy documents presents a viewpoint on the need for more comprehensive collaboration and coordination amongst the actors concerned with its development. Also, the coherence levels across the sectoral documents indicate a need to create awareness and the importance of consistent policy alignment. This could, however, be realised through organised consultation at the horizontal level
3	The Federal Government has consistently extended the target date to end gas flaring due to economic interest, profits derivatives by the IOCs, and economic and personal gains of both the political elites and the lawmakers who regulate and equally control a considerable number of the oil blocks. As Nigeria is heavily dependent on the revenue from oil and gas, any policy that would discourage oil and gas production is deliberately suppressed by the government, lawmakers and the IOCs who benefit massively from continued oil and gas production and gas flaring
4	The Nigerian gas flaring management structure lacks participatory processes. Although there may be stakeholder involvement in gas flaring and venting on paper, local host communities and state governments do not participate in gas flaring management
5	The contradictory role of the NNPC, a parastatal under the Ministry of Petroleum Resources, as a majority shareholder in the joint venture (JV) production sharing contracts (PSC) weakens the potential of the government to monitor, measure and enforce gas flaring regulations

Experiences drawn from a global overview of regulatory practices on gas flaring and venting with relevant lessons and conclusion from international experience and best practices on flaring reduction could prove informative (The World Bank-GGFR 2004). Greater alignment of regulation of gas flaring is possible if greater cross-compliance between competing objectives can be achieved. Global-scale institutional mechanisms such as the Paris Agreement (Erickson and Brase 2019), through the INDC to the global response to climate change, could potentially provide a coordinating approach, even though the institutional landscape at a national level in Nigeria remains complex and evolving. Whilst gas flaring under the PIB is centred around fines under the Act, the economic benefits of NGFCP and PIB cannot be evaluated currently as the current legislation items are yet to formally commence (NASS 2021; NGFCP-DPR 2021). Therefore, although the INDC emphasises voluntary emissions reduction, it could lead to higher ambition in mitigation actions and promote sustainable development.

As unclear power structures within the Nigerian complex MLG system and the lack of policy coherence across sectors negatively influence gas flaring policies’ implementation, the challenges and politics in managing the trade-offs between fossil-fuel-based economic development and low sustainable carbon and social development become even more stark. Economic diversification to avoid heavy dependency on oil and gas revenue and steering policy implementation in gas flaring management in a complex MLG structure provides a key pathway for progress. Greater clarity over the power structures operating across the federal system could support stronger policy coherence, which could, in turn, lead to more effective environmental policy implementation, regulation and enforcement. There is also an urgent need for coordinated horizontal governance to involve a greater array of stakeholder voices, strengthening the various federal institutions to promote policy coherence to reduce the inconsistency across sectors revealed in our analysis. This requires stronger alignment between environmental and other sectoral policy goals, strengthening regional/state powers within the quasi-federal system, and avoiding ‘rural sacrifice’ for geographically remote regions from the dominant political centre. A collaborative policy framework mainstreamed across relevant sectors is crucial to mitigate sectoral policy goal conflicts whilst the Federal Government must mobilise the political will to stop gas flaring. Environmental justice and concerted action to reduce gas flaring may nevertheless be supported through a combination of international pressure, local capacity building and a persistent push for localisation and ‘bottom-up’ governance reforms.

## Supplementary Information

Below is the link to the electronic supplementary material.

Supplementary file1 (DOCX 41 KB)

## Data Availability

The data supporting this study’s findings are available on request from the corresponding author. The data are not publicly available due to their containing information that could compromise the privacy of research participants.
