# Supplementary material for: Gas Flaring in Nigeria: A Multi-level Governance and Policy Coherence Analysis
Source: Anthr. Sci. 2023 Feb 14;2(1):31–47. doi: 10.1007/s44177-023-00045-5 (PMC9927060; doi:10.1007/s44177-023-00045-5)
Supplement: Supplementary file 1 — Supplementary file1 (DOCX 41 KB) [file 44177_2023_45_MOESM1_ESM.docx]

**Expert Survey**

**Title of Project:** A multi-level governance and policy coherence analysis of gas flaring in Nigeria

Preamble: Copyright waiver

**Questionnaire**

Enumerator code……………………………...………………...………….……………………

Name of Respondent ………….………………………………………………………………...

Address………………………………….……………………………………………...…….………………………………………………………………………………………………..........

City/Town/Village: ………………………………………………………………….…………

State……………………………Region: …………………………………….………………...

Date……………………….……Phone number………………………………………...….…

Email Address…………….…………………………………………………………………….

Your organisation: ………………….……………………………………………….………….

Your Position: ………………….……………………………………………….………………

Questionnaire No: ………………….……………………………………………….………….

**Section 1 – Introduction:**

1. What are your main responsibilities in your current role?

………………………………………………………………………………………………………………………………………………………………………………………………………………………………………………………………………………………………………

1. When did you become involved with gas flaring issues?

………………………………………………………………………………………………………………………………………………………………………………………………………………………………………………………………………………………………………

**Section 2 – Administrative structures, relationship between federal institutions and jurisdictional conflicts**

**Relevant Federal institutions tasked with gas flaring and venting management**

1. What role do you think a national oil company (NNPC) should play in the oil and gas industry? ***Please type your preferred number between 1 and 5 in the box where necessary.***

|  | **1=Strongly agree**  **2=Agree**  **3=Undecided 4=Disagree**  **5=Strongly disagree** |
| --- | --- |
| Operator only |  |
| Field management |  |
| Be a regulator/policy maker |  |
| Be vertically integrated |  |
| Midstream (Storage, processing, and transportation of petroleum products) |  |
| Upstream (exploration, drilling, and extraction of oil and gas) and Downstream (refining of petroleum crude oil and the processing and purifying of raw natural gas) |  |
| Influence other government departments |  |
| Investor |  |
| Body to facilitate communication between Government and industry operators |  |
| Redistribution of wealth, fuel subsidies, |  |
| Technology transfer, controls local content |  |
| Should be commercially driven |  |
| Be transparent and independent from government |  |
| Other strategic government objectives |  |

**Administrative structures**

1. In the Nigerian gas flaring management, are there compliance programmes and controls to prevent or detect possible instances of fraud and corruption?

| **1=Yes** | **2=Maybe** | **3=I Do not know** | **4=No Programme** |
| --- | --- | --- | --- |
|  |  |  |  |

4b. If yes, how effective do you believe these programmes are in the Nigerian oil and gas management?

| **1=Very effective** | **2=Somewhat**  **Effective** | **3=Effective** | **4=Unsure** | **5=Not effective** |
| --- | --- | --- | --- | --- |
|  |  |  |  |  |

1. Federal institutions tasked with gas flaring and venting-administrative structures.

|  | **Yes** | **Maybe** | **Unsure** |
| --- | --- | --- | --- |
| **5a**. Are there administrative structures in place to manage gas flaring and venting in Nigeria? |  |  |  |
| **5b.** Strategically, does it matter which of the federal institutions enforce regulations on gas flaring if all the institutions tasked with environmental issues have a common goal of capping gas flaring? |  |  |  |

**Section 3 – Federalism and gas flaring policies, stakeholders/local content, and relevant Federal Government’ institutions**

1. Policy goals/preference

|  | **1=Strongly agree**  **2=Agree**  **3=Unsure**  **4=Disagree**  **5=Strongly disagree** |
| --- | --- |
| **6a.** How well do you agree/disagree that the policy goals and preferences of different groups involved in Nigeria’s gas flaring fit together? |  |
| **6b.** To what extent do you agree/disagree that economic policies dominate environmental policies and concerns over flaring? |  |
| **6c.** Do you think the interests of particular groups have played a role in limiting the implementation of gas flaring policies? |  |
| **6d.** Do you consider the fine of $2 per 1,000 cubic feet of gas for companies which produce 10,000 barrels of oil per day or more, or $0.5 per 1,000 cubic feet for companies producing less than 10,000 barrels a day appropriate as a penalty seeking to reduce flaring? |  |

1. What factors are most likely to influence the decision to implement gas flaring and venting policies/regulations in Nigeria in the next four years?

| **Please, rank from 1-3 according to importance. The number 1 choice has a weight of 1, number 2 choice weights 2, and number 3 choice weights 3.** |  |
| --- | --- |
| Demand for alternative or renewable energy |  |
| Low-cost competition |  |
| Supply chain security |  |
| Disruption of capital markets |  |
| Financing costs |  |
| Downturn in global economy |  |
| Local content |  |
| Inflation |  |
| Oil / Natural gas price |  |
| Energy Input costs |  |
| Fraud and corruption |  |
| Community / social activism |  |
| Regulatory compliance |  |
| Technology |  |
| Inadequacy of basic infrastructure |  |
| Environmental considerations |  |
| People skills |  |

**Federalism and Gas flaring Policies**

1. Do you think the government should decentralise authority to the state and local governments and enable them to legislate and control gas flaring in the various gas flaring and venting states in the Niger Delta?

| **1=Decentralise** | **2=Do not Decentralise** | **3=Unsure** |
| --- | --- | --- |
|  |  |  |

**Stakeholders/Local Content**

1. Do you think the local and state government priorities and politics concerning flaring match those of the national government?

| **1=Yes** | **2=No** | **3=Unsure** |
| --- | --- | --- |
|  |  |  |

9b. If you think they are different, please explain how they differ.

………………………………………………………………………………………………………………………………………………………………………………………………………………………………………………………………………………………………………

1. How significant is strong local stakeholder/local host communities’ involvement in the success of the gas flaring reduction target?

| **1=Very important** | **2=Important** | **3=Not important** | **4=Undecided** |
| --- | --- | --- | --- |
|  |  |  |  |

**Section 4 – International Oil Companies (IOC's), Government policies, and Policy implementation**

1. What percentage of capital expenditure (CAPEX) do you think oil and gas companies should be spending on environmental issues, including gas flaring and venting abatement?

| **Between 5 -10%** | **More than 10%** | **Less than 5%** |
| --- | --- | --- |
|  |  |  |

**International Oil Companies (IOC's) and government policies on gas flaring and venting**

1. Policy change and IOCs influence

| **12a.** Where do you think the balance of power and influence is located between government policy and IOCs? | **Government (%)** | **IOCs (%)** |
| --- | --- | --- |
|  |  |  |

| **12b.**  Do you think the influence of the IOCs weakens or strengthens interest in gas flaring policy implementation, or is it neutral? | **1=Weakens** | **2=Strengthens** | **3=Neutral** |
| --- | --- | --- | --- |
|  |  |  |  |

1. How have gas flaring and venting policies and regulations affected capital project investment decisions by the International Oil Companies (IOCs) over the years?

|  | **1=Significant Impact** | **2=Medium Impact** | **3=No Impact** |
| --- | --- | --- | --- |
| Change oil and gas projects scope |  |  |  |
| Revised oil and gas projects specification |  |  |  |
| Cancelled most oil and gas projects |  |  |  |
| Accelerate most of the oil and gas projects |  |  |  |
| No impact on oil and gas projects |  |  |  |
| Delayed or postponed oil and gas projects |  |  |  |

**Policy implementation**

1. Which of the following do you expect might create difficulties for executing gas flaring reduction strategy policies, and to what extent?

| **1=Strongly agree**  **2=Agree**  **3=Undecided**  **4=Disagree**  **5=Strongly disagree** |  |
| --- | --- |
| Inconsistent and conflicting regulatory framework |  |
| Incoherent policies |  |
| Oil price fluctuations |  |
| Difficulty in securing finance |  |
| Uncertainty in project costs |  |
| Fraud & corruption |  |
| Political uncertainty in country/region |  |
| Lack of skilled labour |  |
| Uncertain in future taxation/royalty payments |  |
| Higher level of project costs |  |
| Oil / Natural gas price |  |
| Energy Input costs |  |
| Fraud and corruption |  |
| Risk of physical safety of staff and Social unrest/activism |  |
| Lack of refining capacity |  |
| Anti-competitive practices |  |
| Reliable/uninterrupted power supply |  |

**Policy implementation**

1. To what extent do other policies’ goals override gas flaring and venting policy and implementation?

| **1=Significantly**  **2=Medium**  **3=No Impact**  **4=Unsure** |  |
| --- | --- |
| Economic Recovery and Growth Plan policy |  |
| Nigeria Vision 20:2020 policy |  |
| Nigeria Economic Sustainability Plan 2020 policy |  |
| Fiscal incentives NNPC policy |  |
| Foreign Direct Investment Regime 2021 policy |  |
| Nigeria economic growth 2020 policy |  |
| Nigerian Investment Promotion Commission policy |  |
| Expansion of Treasury Single Account (TSA) coverage policy |  |
| Agriculture Promotion Policy (2016-2020) |  |
| Nigeria National Energy Policy 2003 |  |
| National Integrated Infrastructure Masterplan policy |  |
| National Policy on Climate Change (NPCC) Adaptation |  |
| National Policy on Environment |  |

1. The federal government of Nigeria control significant shares within the production sharing contract (PSCs) through the Ministry of Petroleum Resources and the Nigerian National Petroleum Corporation. How do you think this affects the federal institutions’ enforcement of gas flaring laws and regulations?

| **1=Not at all** | **2=Somewhat** | **3=Substantially** |
| --- | --- | --- |
|  |  |  |

16b. Do you think it makes the enforcement weaker or stronger?

| **1=Weaker** | **2=Stronger** | **3=Undecided** |
| --- | --- | --- |
|  |  |  |

Are there any other comments you would like to make that may be relevant to the study?

**Thank you for your co-operation.**
